# Transitions in Fertility for Brazilian Women: An Analysis of Impact Factors

**DOI:** 10.1371/journal.pone.0040756

**Published:** 2012-07-13

**Authors:** Regina Madalozzo

**Affiliations:** Department of Economics, Insper Institute of Education and Research, Sao Paulo, Sao Paulo, Brazil; University of Utah, United States of America

## Abstract

The labor participation of Brazilian women has increased during the last few decades. According to the World Bank, the percentage of Brazilian women participating in the labor market rose from 20% in the early 1970s to 65% (for women between 16 and 64 years of age) in 2009. The fertility profile has also changed, the fertility rate has decreased from 6.2 in 1960 to 1.9 in 2009, below the replacement rate, according to the World Bank. This paper will present Brazilian fertility trends during the previous (20^th^) and present (21^st^) centuries and, emphasize the importance of individual profiles for fertility decisions. This work uses Brazilian data from PNAD (*Pesquisa Nacional de Amostra por Domicilios*) to understand the cause of this relationship and to predict the consequences of these choices on women’s economic development.

## Introduction

As in many countries around the world, Brazilian women have engaged more actively in the labor force over the last three decades than in the past. In 1980, only 40.6% of women between 16 and 64 years of age were employed or actively seeking employment in Brazil. In 2009, this number increased to 64.3%, which, according to the World Bank [1], is on par with Spain (63.1%) and France (65.5%). At the same time, male labor participation decreased slightly, from 88.20% in 1980 to 85.20% in 2009. Many studies have discussed this increase in female labor participation and have offered a variety of explanations. The majority of these studies are designed to understand female labor participation in a separate context from fertility decisions. For instance, [2] use Pesquisa Nacional de Amostra por Domicílios (PNAD) to estimate the elasticity of female labor supply in Brazil using various empirical approaches, and they found the expected relation: an increase in real wages leads to an increase in labor supply. [3], [4] analyze the trend in female labor supply using PNAD data for the 1970s, 1980s and 1990s. By modeling fertility, health and labor participation in Cameroon, [5] concluded that although labor participation reduces fertility rates, having a child increases a woman’s propensity to work. [6] use evidence from different countries throughout the world to motivate a theoretical model that suggests that the increase in labor participation was motivated by the decline in wage discrimination against women and that the reduction in child mortality reduced fertility. [7] use Colombian data to estimate labor supply determinants for women with and without children. Their conclusions highlighted the importance of female education, marital status and family income. [8] use Brazilian census data to infer the importance of economic and social development for aggregated fertility rates. These authors found a strong relationship between these variables.

In this paper, we use the changes in fertility indicators to motivate the determination of the characteristics that are most likely to affect individual choice regarding how many children to have. We use data from *Pesquisa Nacional de Amostra por Domicílios* (PNAD) elaborated by the *Instituto Brasileiro de Geografia e Estatística* (IBGE) to quantify the behaviors of different cohorts with respect to this decision. After capturing the actual fertility trend, we focus our study on PNAD 2009 data and analyze the factors that influence the choice of how many children to have. Focusing on two different sub-samples (women between 18 and 45 years of age and women between 46 and 64 years of age), we were able to discover significant changes in the individual variables that impact this choice. While the importance of education and marital status regarding fertility choice is maintained, living in urban areas, geographical region of residence and labor participation decrease the influence of these two variables. This study also provides a description of fertility trends and labor market participation in Brazil. Section 3 describes the data from PNAD 2009 and the methodological approach to the problem, and Section 4 discusses the results. Finally, the conclusion summarizes our findings.

## Analysis

Female labor supply in Brazil has increased significantly in recent decades [3], [4]. Since the late 1980s, perhaps because of the greater number of women in the labor market, revisions to women’s legal rights have rendered the labor environment as less discriminatory against women. The following examples are illustrative [9]:

- The 1988 National Constitution increased the period of maternity leave from 3 months to 120 days (Law no. 10.421– April 15, 2002– extended the benefit to adoptive mothers);
- Law no. 9.029 (April 13, 1995) revoked the right of employers to request pregnancy tests before hiring a woman;
- Law no. 10.224 (May 15, 2001) addresses sexual harassment issues and stipulates a sentence of one to two years of detention for individuals who are tried and found guilty of sexual harassment.

**Figure 1:**
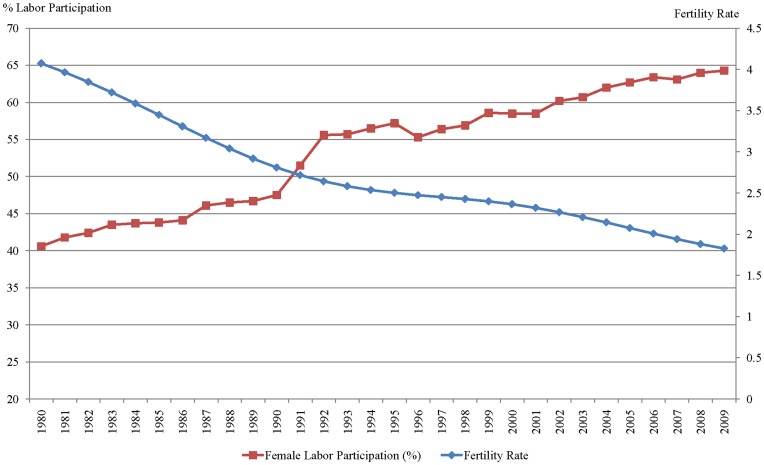
Fertility Rate and Female Labor Force Participation in Brazil (1980–2009). Source: World Bank.

With respect to fertility rates [1], the Brazilian population had 6.2 births per woman in 1960. By 2009, the fertility rate had decreased to 1.9 births per woman, which is below the replacement rate. The replacement rate is approximately 2.33 children per woman: each child replaces one parent, and the additional 0.33 accounts for the mortality rate. Although mortality rate varies by country, the replacement rate is considered to be 2.1 children per women in most industrialized countries.

The comparison between the decrease in fertility rates among Brazilian women and the increase of females in the Brazilian labor market, as depicted in Figure 1, is a particularly interesting analysis. Data from 1980 to 2009 reveal that the steady decrease in fertility was accompanied by a reasonably steady increase in female labor participation. The period of the most rapid increase in female labor participation occurred between 1980 (40.6%) and 1992 (55.6%), which coincides with a more rapid decline in the fertility rate, as the number of children per woman dropped from 6.2 in 1980 to 2.6 in 1992. [6] posit that the reduction in mortality rates also had a significant influence on this relationship. That is, as people live longer, they invest more in human capital, which, in turn, results in an increase in female labor participation and a reduction in the pay gap between genders. [5] use fertility, health and labor supply data to estimate the relationship between labor participation and fertility in Cameroon. Their results suggest a strong relationship between fertility and health conditions, and they not that engagement in the labor market decreases fertility rates, whereas having children increases the chances of female labor participation. These studies contribute to the idea that fertility has a strong relationship with female labor participation; however, the causality of this relationship remains unknown.

**Figure 2:**
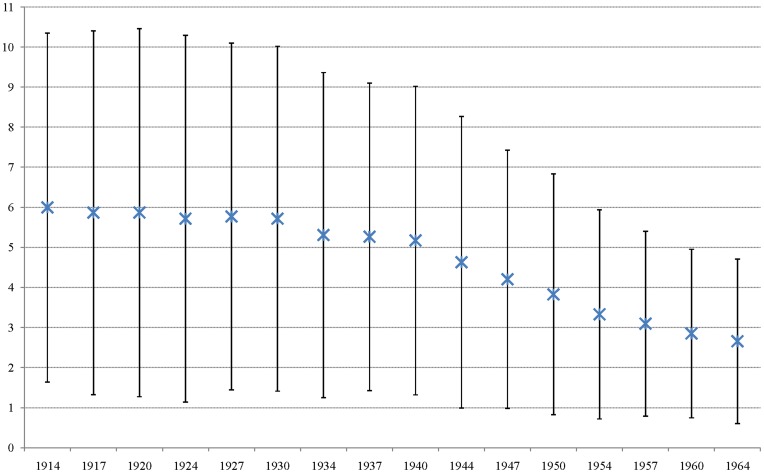
Average Number of Children per Woman by Cohorts (1914–1964). Source: PNAD 1992 to 2009. Author’s tabulation.

Accordingly, the objective of this paper is to explore the relationship between fertility, female labor participation, and several other factors. We used the PNAD microdata to analyze this trend. Using data from 1992 to 2009, we selected a sample of women who were at least 45 years of age to understand the fertility profiles of distinct cohorts. The reproductive life of a woman is typically concluded at the age of 45. We choose this age to obtain more precise estimates of the number of children that each woman in the sample had during her lifetime. Figure 2 shows the results. For women born in 1914, the average number of children was approximately 6, with large variation. From 1914 until 1930, the fertility rate slowly declined. The cohorts born between 1934 and 1940 similarly showed a decline in the number of children in the family with an average of 5.2 children per women. After these cohorts, the fertility decrease was more pronounced. For women born in 1944, the average number of children per woman was 4.62, and, for the female cohort born in 1965, the average number of children decreased to 2.66. As expected, the reduction in the average number of children per women, which was also found in the cohorts born in 1947, 1950 and thereafter, also reduced the variation in the results. The last cohort that was examined was that of women born in 1964, when the average number of children was 2.66 per woman, and the variation in this sample was smaller than in the preceding cohorts.

**Figure 3:**
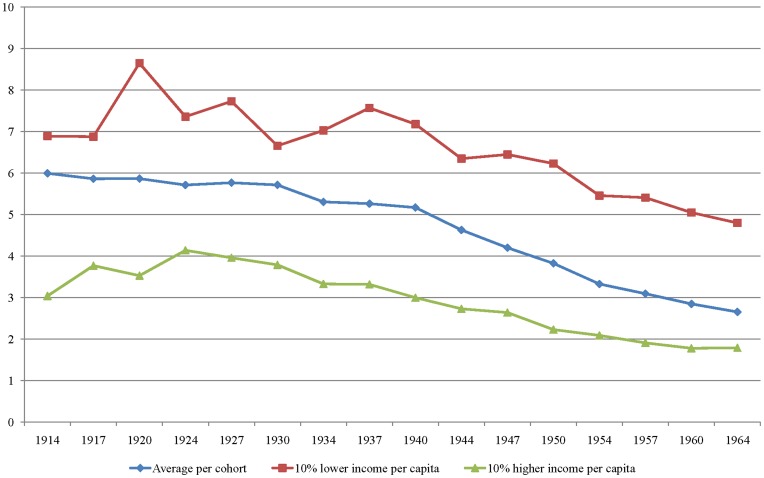
Number of Children and Income Centile by Cohorts (1914–1964). Source: PNAD 1992 to 2009. Author’s tabulation.

Because family income is expected to influence the number of children in a family, we analyze the same cohorts by income percentiles in Figure 3. This figure shows that higher per capita income is associated with a lower number of children per women. For the lowest 10^th^ percentile of income, the 1914 cohort had 6.89 children per women. For the younger cohort, born in 1964, we observed a reduction to 4.8 children per women. For those with the highest 10^th^ percentile of income, the decrease in fertility was subtle. For the cohort of women who were born in 1914, the average number of children per woman was 3.04. For women who were born in 1964, this statistic was 1.79 per woman. Thus, the rapid decrease in the Brazilian fertility rate was the result of an enormous decrease in the fertility rates of poor families.

Fertility issues may be analyzed using the total fertility rate, an aggregate measure for the country or for some determined region, or microdata. Aggregate, macro-level data are more convenient for forecasting because microdata contain finite information pertaining to individuals. As such, many of the important factors that determine fertility choices are not explored in large-sample questionnaires. In this paper, our goal is not to forecast fertility outcomes; rather, we aim to understand individual characteristics that influence these outcomes.

Considering this purpose, we chose the most recent PNAD available (2009) and restricted our sample to women between 18 and 64 years of age. Table 1 presents the demographic information for our sample. Because many women in the sample may not have completed their lifetime reproductive period, we also divided this sample into two groups: women between 18 and 45 years of age who may not have completed the reproductive period in their live, and women between 46 and 64 years of age who are expected to have concluded their fertility profile. These two distinct samples (18 to 45 and 46 to 65 years of age) may also contribute to the analysis of possible changes in the factors that influence a woman to have an additional child. An interesting analysis was made by [10] with a stronger age restriction: 20 to 24 years old and comparing African-Americans and Whites. Our analysis relates to this study, but employs a broader sample.

**Table 1:** Demographic Information Pertaining to Women in Brazil (2009).

	18–64 years old	18–45 years old	46–64 years old
Age	38.16(13.02)	30.94(7.99)	54.21(5.58)
Number of children	2.08(2.18)	1.51(1.59)	3.35(2.72)
Marital Status (%):			
Married	49.07	44.85	58.46
Separated/*Desquitada*	3.26	2.47	5.02
Divorced	3.82	2.64	6.47
Widowed	4.98	1.21	13.37
Single	38.87	48.83	16.68
Ethnicity (%):			
White	49.76	48.33	52.94
Black/*Pardo*	49.55	51.03	46.26
Oriental	0.46	0.42	0.55
Indigenous	0.23	0.22	0.25
Education (%):			
No education	8.04	4.65	15.54
Incomplete Primary	31.28	25.64	43.77
Complete Primary	15.85	17.61	11.96
Secondary	34.8	41.73	19.04
Tertiary or Higher	10.16	10.37	9.69
Region (%):			
South	14.98	14.31	16.47
Southeast	42.79	41.23	46.26
Central	7.41	7.70	6.77
North	7.47	8.20	5.84
Northeast	27.35	28.56	24.66
Labor Force Participation (%)	65.73	71.37	53.18
Living in Urban Area (%)	86.25	86.31	86.18
Per Capita Family Income	693.83(1,172.00)	612.57(965.60)	874.84(1,517.01)
Number of Observations	126,643	88,328	38,315

Source: PNAD 2009, IBGE. Author’s tabulation.

Notes: Parentheses denote the standard deviation.

The average age of our total sample was 38.16 years. On average, each woman had 2 children. An indication that the younger sample had not completed their fertility outcome is that these women had, on average, 1.51 children, whereas the women in the older sample had 3.35 children each, on average.

Concerning marital status, 49.07% of all participants in the study were married and 38.87% of the participants were single. The profile of marital status for the younger cohort found that 44.85% were married and 48.83% were single, which varied significantly from the marital status of the older cohort, in which 58.46% were married, 16.68% were single and 13.37% were widowed.

With respect to ethnicity, the sample was equally divided between Whites and Blacks/*Pardo* (in Brazil, we have one specific designation for the self-declaration of race for “Black” and a different term for a mixed race between White and Black, *Pardo*; the literature also denominates *Pardo* as “Mulatto”); 49.76% of the participants were White, 49.55% were Black/*Pardo* and less than 1% were Oriental or Indigenous. The race of the sub-samples differed slightly; the younger sub-sample had a slight majority of Black/*Pardo* (51.03%) women and the older sub-sample had a slight majority of White (52.94%) women.

As other studies have reported [11], [12], education profile in Brazil changed significantly, especially for females, within the time period of interest. Women who belong to the older sub-sample were less educated than those in the younger cohort. The majority of the women in the older sub-sample had, at most, an incomplete primary school education (59.31%), whereas only 30.29% of the younger sub-sample reported such minimal education. The majority of the younger sub-sample had completed at least some secondary school education (52.10%).

Following the geographical distribution of the population in Brazil, the sample was highly concentrated in the southeast region (42.79%), followed by the northeast (27.35%) and the southern (14.98%) regions. The northern and central regions comprised only 14.88% of the total sample. Moreover, 86.25% of the sample lived in urban areas. There was little variation in this percentage between sub-samples.

Labor market participation varies with age, especially when analyzing female outcomes [13]. As such, labor participation was greater for the women in the younger sub-sample (71.37%) and smaller for those in the older sub-sample (53.18%). On average, 65.73% of the women between 18 and 65 years of age were working or actively seeking work in 2009.

Finally, our sample had a per capita family income of R$693.83. This value was insignificantly higher for the older sub-sample (R$874.84) than for the younger sub-sample (R$612.57).

In the next step of our study, we examine how these individual characteristics may influence the choice to have an additional child. One important characteristic that our data do not contain is “health condition”. According to [14], this variable should have strong effect on the outcome, but the absence of this information is a restriction of our dataset. To this end, we used an ordinary least squares regression model with robust standard errors and an analysis of factor contribution.

The main model is described in Equation (1):
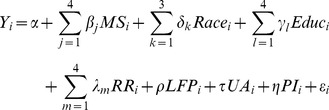
(1)


where

Y_i_ is the number of children that each woman had to date;

MS represents the marital status of each woman (the comparison group is single);

Race is a dummy variable (the comparison group is White);

Educ is an education dummy (the comparison group is “No formal education”);

RR is a dummy for region of residence (the comparison group is southeast);

LFP is a dummy that takes the value of 1 when an individual participates in the labor market and zero otherwise;

UA is a dummy that takes the value of 1 when an individual lives in an urban area and zero otherwise; and

PI is the per capita family income (a continuous variable).

The estimated coefficients of Equation (1) represent the direct effect of each characteristic on the number of children. However, we also aim to understand the percentage of influence of each personal attribute on the number of children that a woman chooses to have. Accordingly, we also calculated the factor contribution using a standardized regression. The standardized regression closely follows Equation (1) but utilizes all of the parameters that were standardized over the variance that was set to 1, as shown in Equation (1). By standardized variable, we mean that each observation is diminished by the variable mean and divided by its variance. For instance, standardizing Y would be accomplished through the calculation 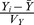, where Y is the variable, 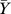
is the mean of Y, and VY is the variance of Y. The estimated coefficients express the degree to which each variable influences the outcome based on the standardization.

Additionally, we preferred to report the percentage of influence of each variable on the response variable. Therefore, for each regression, we totaled the estimated standardized coefficients and calculated the percentage impact of each variable on the number of children per woman. In the next section, we present and discuss these results.

## Results

In this section, we examine the results from our estimated model. Table 2 presents the coefficients for Equation (1) that were estimated for our three samples: the entire sample of women between 18 and 64 years of age, the younger sub-sample (18 to 45 years of age), and the older sub-sample (46 to 64 years of age). Although the estimated coefficients are closely related, the analysis of the impact of each variable on the response variable varies.

**Table 2:** Estimations and coefficients of partial contributions to the “number of children” - Equation (1).

	18–64 years old	18–45 years old	46–64 years old
Marital Status:			
Separated/*Desquitada*	1.40^***^[4.25]	1.06^***^[4.60]	1.32^***^[4.45]
Divorced	1.41^***^[4.65]	1.11^***^[4.98]	1.22^***^[4.62]
Widowed	2.00^***^[7.44]	1.37^***^[4.17]	1.61^***^[8.39]
Married	1.22^***^[10.44]	0.90^***^[12.54]	1.36^***^[10.27]
Race:			
Black/*Pardo*	0.23^***^[1.95]	0.16^***^[2.29]	0.45^***^[3.41]
Oriental	−0.09[0.11]	−0.04[0.07]	−0.31^*^[0.35]
Indigenous	0.34^**^[0.28]	0.34^***^[0.46]	0.38[0.29]
Education:			
Incomplete Primary	−1.33^***^[10.56]	−0.53^***^[6.42]	−1.36^***^[10.37]
Complete Primary	−2.27^***^[14.19]	−1.34^***^[14.19]	−2.06^***^[10.26]
Secondary	−2.70^***^[22.03]	−1.73^***^[23.78]	−2.48^******^[14.92]
Tertiary or Higher	−2.69[13.92]	−1.68^***^[14.27]	−2.63^***^[11.95]
Region (%):			
South	0.07^***^[0.44]	0.05^**^[0.48]	0.12^***^[0.68]
Central	0.21^***^[0.96]	0.19^***^[1.44]	0.34^***^[1.32]
North	0.72^***^[3.12]	0.50^***^[3.69]	1.48^***^[5.09]
Northeast	0.31^***^[2.37]	0.08^***^[0.97]	1.04^***^[6.91]
Labor Force Participation (%)	−0.09^***^[0.71]	0.05^***^[0.62]	−0.14^***^[1.04]
Living in Urban Area (%)	−0.21^***^[1.25]	−0.4^*^[0.43]	−0.78^***^[4.11]
Per Capita Family Income	−0.00007^***^[1.34]	−0.0002^***^[4.60]	−0.0006^***^[1.58]
Constant	3.28^***^	2.23^***^	3.97^***^
R-squared (%)	31.32	27.80	25.82
Number of Observations	124,175	86,473	37,702

Source: PNAD 2009, IBGE. Author’s tabulation.

Notes:

i) The brackets indicate the contribution (in percentage points) of each variable to the total explained potential of the estimated equation.

ii) The significance of the coefficients is labeled as follows: * represents a p-value<0.05, ** represents a p-value<0.01, and *** represents a p-value<0.001.

Marital status significantly affected the number of children that a woman chose to have. The baseline category for analysis was “single”, and all of the other categories have a significant positive effect on the number of children; thus, the results indicate that being in a relationship (formal or informal) highly increased the chances of having a child, as expected. When analyzing the standardized coefficient, being married had a greater impact than other marital statuses, explaining 10.44% of the variation in having an additional child. Overall, the impact of relationship status on the number of children represented 26.78% of the total variation in the response variable.

Race also played a significant role on the number of children, but its influence was smaller. The baseline category for this analysis was “White”, and we observe that both “Black/*Pardo*” and “Indigenous” had more children than the baseline group. For the older sample, being “Oriental” decreased the chances of having an additional child. Race was a more important factor for the older sub-sample, representing 4.04% of the total variation in the dependent variable, while it represented 2.34% for the total sample and 2.81% for the younger sub-sample.

As other studies have also reported [15], [16], education plays a major role with respect to fertility issues. Each additional educational level greatly reduces the projected number of children that a woman chooses to have. For the younger sub-sample, education explained 58.67% of the total variation in the dependent variable. The older sub-sample had a lower corresponding percentage (47.50%), but education still exerted the greatest impact on the number of children per woman.

The region of residence also explains some of the variation in the response variable. Living in the southeast region was associated with an expected reduction in the number of children per woman. The region of residence had a greater influence for the older sub-sample, for which this factor accounted for 14% of the total variation in individual fertility, than for the younger sub-sample, for which it accounted for only 6.58% of the total variation. Living in an urban area played a minor role on the number of children (approximately 1% for the total sample). This variable had a greater effect among the older sub-sample, for which 4.11% of the total variation on the dependent variable could be explained by residence in an urban area. With the decreased fertility rates in all regions, this variable is likely to have less influence on future generations/cohorts.

[Thightest]Whether a woman was engaged in the labor market did not strongly affect the number of children that she chose to have, although this factor significantly affected other areas. The difficulty with this control variable is that the women in our sample were at different points in their lives. Younger women may not have entered the labor market by the time of the interview, whereas older women may have departed from the labor market at that time.

In the present study we used cross-sectional data, because there is a scarcity of panel data in Brazil. One available panel data set would be Pesquisa Mensal de Emprego (PME), from IBGE, but this panel covers only for a year, with the sample being interviewed under a4-8-4 scheme. This time duration would not assist in resolving the problem of capturing labor participation during the period in which these women from different cohorts had children. Apart from the cohort analysis, [17] analyzes maternity and labor engagement in Brazil. However, using a cross-sectional data, as we do, presents a problem for interpretation. For the younger sub-sample, labor force participation increased the chances of having an additional child, whereas the opposite effect was found for the total sample and the older sub-sample. However, the contribution of this factor was modest in the total variation: only 0.71% for the total sample and slightly greater than 1% for the older sub-sample.

Finally, we analyzed the impact of family income on fertility. Figure 3 indicates that income significantly affected the number of children that a woman had, and our regression showed that a higher per capita family income was associated with a lower probability of having an additional child. However, the impact of family income differed by sub-sample. For the older sub-sample, the effect of family income on the number of children accounted for only 1.58% of the total variation. However, when we analyzed the younger sub-sample, we found that 4.60% of the total variation could be explained by this variable. Thus, education and income profile play a greater role on fertility matters today than in the past. It is important to note that we did not control for personal income, we only controlled for family income. Therefore, female income, when present, was included but did not determine the results. For an interpretation of this stronger effect on younger cohorts, we examined Figure 3. Although the lower-income women showed a substantial decrease in the number of children, the higher-income women also reduced their target fertility. The average number of children was closer to the fertility outcomes of the higher-income women than those of the lower-income women.

## Discussion

Fertility rates have decreased during the last several decades in the majority of countries, and this decrease was particularly significant in Brazil. Compared with 6.2 children per woman in 1980, Brazil had a fertility rate of 1.9 children per woman in 2009. An increase in education, a decline in mortality rates and an increase in female labor participation are possible explanations for the declining fertility rates. These findings are consistent with previous results. [8] used Brazilian census data from 1960 to 1991 and found the same negative effect of education on fertility. Although they used Colombian data, [7] reached the same conclusion with regard to education and income. The current work explored these features in the context of cross-sectional data. The PNAD 2009 enabled a snapshot analysis of the factors that influenced the decision of a woman to have additional children.

Using a framework of an ordinary least squares regression and the variation of standardized coefficients, we concluded that the role of education and marital status had stronger effects on this choice, independent of the cohort that was analyzed. Nevertheless, family income plays a greater role among younger cohorts than in the past, and income may have stronger consequences in the future than are currently anticipated. Because higher-income mothers generally seem to choose smaller family sizes and because lower-income mothers often seem to prefer large families, income concentration may become more severe.

As a policy recommendation, this study suggests that continuing to promote higher education for women will have a powerful effect on fertility rates. As [6] posit, the increase in human capital investment has the effect of reinforcing female engagement in the labor market and reducing the gender wage gap. It is more expensive for highly educated women to abandon their careers or postpone their work than for less educated women; therefore, more highly educated women make greater investments in the labor market and choose to have fewer children during their lifetime. For Brazilian public policy makers who want to continue to reduce the income disparity between families – and genders-, increasing female education will target the problem of qualification for labor market engagement and reduce family size among the low-income population.
